# Differential Impact of Social Cohesion in the Lens of U.S. College Students with different Sexual and Gender Identities on their Mental Health during the COVID-19 Pandemic

**DOI:** 10.1192/j.eurpsy.2023.323

**Published:** 2023-07-19

**Authors:** Y. Lin, W. Deng, H. Xie

**Affiliations:** 1 Psychiatry and Behavioral Science, Virginia Tech Carilion School of Medicine, Roanoke; 2 Psychology, Yale University, New Haven; 3 Publich Health, University of Wisconsin, Milwaukee, United States

## Abstract

**Introduction:**

Sexual and gender minority (SGM) college students endorsed higher psychological distress and worsened mental health outcomes than their cisgender heterosexual peers. Such disparity is exacerbated during the COVID-19 pandemic, during which SGM youth may be sent home to unaccepting environments or presented with fewer healthcare options. The “Black lives matter (BLM)” and “Anti-Asian Hate” also exposed college students disproportionally to more witnessed discrimination and poorer social cohesion, which in turn, might negatively affect the mental health outcomes.

**Objectives:**

The present study aims to explore the mental health outcome profile within SGM college students by (1) identify mental health disparities across different sexual and gender identities and (2) evaluating the impacts of discrimination, social cohesion and other factors on mental health outcomes of college students with different sexual and gender identities.

**Methods:**

The study utilizes the 2020-2021 Healthy Minds Study data with 139,470 college students across 60 U.S. campuses. Multivariable regression models are built with minority status to predict mental health outcome (depression, anxiety, and suicidal ideation).

**Results:**

SGM students reported higher symptoms of depression, anxiety, and suicidal ideation. Besides, SGM individuals having experienced or witnessed discrimination or hostile behaviors due to their race/ethnicity also showed worse mental health outcomes. Noted, perceived stronger social cohesion is a protective factor for lower depression (OR: 0.59; 95%CI: 0.45, 0.78) and anxiety (OR: 0.69; 95%CI: 0.51, 0.93) symptoms in SGM, while perceived weaker social cohesion is a risk factor for depression (OR: 1.37; 95%CI: 1.14, 1.64) and anxiety symptoms (OR:1.32; 95%CI:1.09-1.59) in cisgender heterosexual individuals.

**Conclusions:**

These findings acknowledge the negative impact of discrimination on mental health, highlight the importance of recognizing social cohesion affect differently in SGM and their peers, and enhance the understanding of differential impact of social cohesion to inform public policy and early intervention in vulnerable populations during COVID-19 pandemic.

**Disclosure of Interest:**

None Declared

